# Tuning the Stiffness Balance Using Characteristic Frequencies as a Criterion for a Superconducting Gravity Gradiometer

**DOI:** 10.3390/s18020517

**Published:** 2018-02-08

**Authors:** Xikai Liu, Dong Ma, Liang Chen, Xiangdong Liu

**Affiliations:** MOE Key Laboratory of Fundamental Physical Quantities Measurement & Hubei Key Laboratory of Gravitation and Quantum Physics, School of Physics, Huazhong University of Science and Technology, Wuhan 430074, China; liuxikai@hust.edu.cn (X.L.); madong@hust.edu.cn (D.M.); liangchen@hust.edu.cn (L.C.)

**Keywords:** superconducting gravity gradiometer, common-mode rejection, stiffness balance, response function measurement, characteristic frequency

## Abstract

Tuning the stiffness balance is crucial to full-band common-mode rejection for a superconducting gravity gradiometer (SGG). A reliable method to do so has been proposed and experimentally tested. In the tuning scheme, the frequency response functions of the displacement of individual test mass upon common-mode accelerations were measured and thus determined a characteristic frequency for each test mass. A reduced difference in characteristic frequencies between the two test masses was utilized as the criterion for an effective tuning. Since the measurement of the characteristic frequencies does not depend on the scale factors of displacement detection, stiffness tuning can be done independently. We have tested this new method on a single-component SGG and obtained a reduction of two orders of magnitude in stiffness mismatch.

## 1. Introduction

Over the past few decades, the airborne rotating gravity gradiometer has been proven to be a powerful tool in geodetic surveys and mineral exploration [1,2,3,4]. Spurred by its successful applications, a number of new technologies have been developed with the motivation to update the resolution in the future for airborne gravity gradients measurement [5,6]. Among them, the superconducting gravity gradiometer (SGG) is extensively considered as one of the candidates with the most potential [7]. It should be noted that we only considered the airborne SGG in the following. A number of pioneering works on SGG have been carried out in Paik’s group in Maryland [8,9,10,11,12,13]. These works have distinctly shown its advantages as a next-generation gradiometer. First, the SGG makes use of the Meissner effect to levitate superconducting test masses, thus forming superconducting spring oscillators. Benefiting from the cryogenic environment, the oscillators show ignorable thermal noise. Second, the SGG uses superconducting interference devices to detect the displacements of oscillators, resulting in extremely low instrumental noise. The above two features jointly enable the SGG to be the most unique instrument with a fundamental noise floor lower than 1E/√Hz (1E = 10^−9^ s^−2^) in the laboratory today [8].

However, lower fundamental noise does not mean better resolution for airborne measurements. In the notorious aviation environment, the vibration level of the platform is higher than that in the laboratory by 4–6 orders of magnitude [14]. The platform vibration coupling noise eventually decides the measurement resolution, as the fundamental noise only acts as an insurmountable physical limit for the instrument. Therefore, the onboard measurement conditions impose at least two technological requirements on the development of airborne SGG, i.e., an extremely large common-mode rejection ratio and an extremely low cross-coupling coefficients, which guarantee that the measurements are immune to the vibration of the platform in sensitive and non-sensitive degrees of freedom, respectively [12]. To depress the vibration coupling noise to an acceptable level, a number of technological problems need to be solved for SGG [13]. Nevertheless, this article only deals with the tuning problem concerning common-mode rejection.

To reject the common-mode noise, the rotating accelerometer gravity gradiometer ingeniously introduces regular common-mode modulation acceleration by rotating the installation disc [15]. With this modulation, the scale factors of the accelerometers are continuously and precisely balanced during the measurement, and the gravity gradient signals are demodulated out with a large common-mode rejection ratio [16]. Unfortunately, it is quite difficult to apply the rotation strategy to the SGG due to its incompatibility with the cryogenic environment. Instead, the SGG adopts an off-line method to tune common-mode balance [8]. This is to say, the instrument should be finely tuned to the optimum state before measurement. This method is feasible, as the tuned working state can be perfectly preserved thanks to the high stability of supercurrents in the SGG [7]. Theoretical analysis has revealed that, to achieve a full-band common-mode balance, both the stiffness and the scale factor of displacement detection should be precisely matched. Normally, the SGG tunes the stiffness to balance by adjusting the supercurrents in levitating coils [12]. These details are illustrated in Appendix A. More choices exist to tune the scale factor. One can adjust the supercurrents in the detecting coils in the same way as stiffness tuning, but this method gives rise to one problem. As the coils interact with the test masses, the tuning of the scale factor and that of the stiffness are entangled and severely interfere with each other. Another option is to keep the supercurrents in the displacement-detecting coils constant, tuning the scale factor to balance using an electronic device or via data processing. For tuning common-mode balance, the stiffness balance is essentially important for two reasons. First, it is a prerequisite for full-band common-mode balance. Second, as will be clarified in the following section, badly matched stiffness parameters will result in a high-amplitude output at the oscillator’s resonance frequencies. These signals cannot be reduced by tuning the scale factors. They are not only useless for gravity gradient measurement, but also severely compress the dynamic range of the instrument.

However, the question of how to precisely tune the stiffness remains unsolved. The difficulty mainly comes from the lack of a reliable criterion to evaluate the tuning effect. In [8], Moody and his colleagues proposed a wide-band stiffness-tuning method. In this method, assuming the scale factors of displacement detection for two oscillators are identical, the supercurrents are finely adjusted to null the response to the applied common-mode acceleration. However, this method does not work if the scale factor of displacement-detecting is badly matched. In this case, the disappearance of response only indicates that the product of the transfer factor from acceleration to displacement and that of displacement-detecting is well matched. In this way, a common-mode balance can be realized only at a single frequency point rather than over the full-band, since the balance conditions vary significantly with frequency. Therefore, the disappearance of response cannot be used as a criterion for stiffness balance if the scale factor is not precisely matched.

In this paper, a new scheme is proposed to tune the stiffness. In this scheme, the characteristic frequencies of the SGG oscillators are adopted as the criterion. Since these frequencies do not depend on the scale factors of displacement-detecting, the criterion works even if the scale factors are badly matched. Furthermore, once the characteristic frequencies have been measured, one can immediately know how far away the working state deviated from the ideally matched state. Through simple calculations, one can easily estimate how much the currents should be adjusted in the next step. The tuning scheme was successfully applied to a single-component SGG, where the stiffness balance was improved by about two orders of magnitude.

## 2. Theoretical Analysis

The working principles of the SGG have been described in detail in a number of references [8,9,10,11,12,13]. Here, we provide a brief introduction. Taking a T_zz_ single-component SGG as an example, a superconducting vertical spring oscillator is constructed by levitating a test mass in the superconducting Meissner state using a current-carrying superconducting coil. A pair of oscillators are installed along the vertical direction at a certain distance. For the convenience of optimizing the performance, the levitating superconducting coils of two oscillators are incorporated into the same superconducting circuit. Governed by the flux conservation law of superconducting loops, the motions of two oscillators are coupled by the superconducting circuit. These two coupled oscillators can be modeled as a two-degree-of-freedom vibration system. The vibration can be decomposed into two normal modes: the common mode and the differential mode. The differential-mode displacement is detected as a gravity gradient signal. By coupling the two oscillators together through the superconducting circuit, the differential-mode stiffness can be designed to be much smaller than that of the common mode; consequently, the instrument sensitivity and the ability to reject common-mode noise can be improved.

The dynamic equations of the oscillators are written as

(1) $$\left\{ \begin{array}{l} m_{1}{\ddot{x}}_{1}+c_{1}{\dot{x}}_{1}+K_{10}x_{1}+K_{12}x_{2}=m_{1}a_{1}\\ m_{2}{\ddot{x}}_{2}+c_{2}{\dot{x}}_{2}+K_{21}x_{1}+K_{20}x_{2}=m_{2}a_{2} \end{array}\right.$$

where *m*_1_ and *m*_2_ are the masses; *x*_1_ and *x*_2_ are the displacements of the test masses; *a*_1_ and *a*_2_ are the input accelerations that act on the test masses; *c*_1_ and *c*_2_ are the damping coefficient of the spring oscillators. *K*_10_, *K*_20_, *K*_12_ and *K*_21_ are the stiffness coefficients, which are determined by the parameters of the superconducting circuit and the injected supercurrents. Their expressions are given in Appendix A. The stiffness coefficients *K*_12_ and *K*_21_ are always identical because of the exchange symmetry of the superconducting circuit with respect to the test masses. By defining

(2) $$\left\{ \begin{array}{l} a_{c}=\frac{a_{1}+a_{2}}{2}\\ a_{d}=a_{1}-a_{2} \end{array}\right.$$

Equation (1) can be solved to produce

(3) $$\begin{array}{l} x_{1}\left(\omega \right)=\frac{\left(-\omega^{2}+\frac{j\omega c_{2}}{m_{2}}+\frac{K_{20}}{m_{2}}+\frac{K_{12}}{m_{1}}\right)a_{d}\left(\omega \right)+\left(-\omega^{2}+\frac{j\omega c_{2}}{m_{2}}+\frac{K_{20}}{m_{2}}-\frac{K_{12}}{m_{1}}\right)a_{c}\left(\omega \right)}{\left(-\omega^{2}+\frac{j\omega c_{1}}{m_{1}}+\frac{K_{10}}{m_{1}}\right)\left(-\omega^{2}+\frac{j\omega c_{2}}{m_{2}}+\frac{K_{20}}{m_{2}}\right)-\frac{{K_{12}}^{2}}{m_{1}m_{1}}}\\ x_{2}\left(\omega \right)=\frac{-\left(-\omega^{2}+\frac{j\omega c_{1}}{m_{1}}+\frac{K_{10}}{m_{1}}+\frac{K_{12}}{m_{2}}\right)a_{d}\left(\omega \right)+\left(-\omega^{2}+\frac{j\omega c_{1}}{m_{1}}+\frac{K_{10}}{m_{1}}-\frac{K_{12}}{m_{2}}\right)a_{c}\left(\omega \right)}{\left(-\omega^{2}+\frac{j\omega c_{1}}{m_{1}}+\frac{K_{10}}{m_{1}}\right)\left(-\omega^{2}+\frac{j\omega c_{2}}{m_{2}}+\frac{K_{20}}{m_{2}}\right)-\frac{{K_{12}}^{2}}{m_{1}m_{1}}} \end{array}.$$

Here, $a_{c}$ is the motion acceleration of the platform, and $a_{d}$ is the differential acceleration resulting from the gravity gradient. From Equation (3), the differential displacement of the test masses can be expressed as

(4) $$\begin{array}{l} x_{1}\left(\omega \right)-x_{2}\left(\omega \right)=\\ \frac{\left(-2\omega^{2}+\frac{j\omega c_{1}}{m_{1}}+\frac{j\omega c_{2}}{m_{2}}\frac{K_{20}}{m_{2}}+\frac{K_{10}}{m_{1}}+\frac{K_{12}}{m_{2}}+\frac{K_{12}}{m_{1}}\right)a_{d}\left(\omega \right)+\left(\frac{K_{20}}{m_{2}}-\frac{K_{12}}{m_{1}}+\frac{j\omega c_{2}}{m_{2}}-\frac{K_{10}}{m_{1}}+\frac{K_{12}}{m_{2}}-\frac{j\omega c_{1}}{m_{1}}\right)a_{c}\left(\omega \right)}{\left(-\omega^{2}+j\omega \frac{c_{1}}{m_{1}}+\frac{K_{10}}{m_{1}}\right)\left(-\omega^{2}+j\omega \frac{c_{2}}{m_{2}}+\frac{K_{20}}{m_{2}}\right)-\frac{{K_{12}}^{2}}{m_{1}m_{1}}} \end{array}.$$

In the SGG, the differential displacement is detected as a gravity gradient signal.

It can be clearly seen that, if the parameters of the oscillators are not matched, the platform acceleration will enter into the expression of the differential displacement. To achieve an excellent full-band common-mode balance, the correlated parameters should be elaborately adjusted to minimize the dependence of differential displacement on the platform acceleration. It is easy to derive two conditions from Equation (4) for an ideal balance, which are

(5a) $$\frac{K_{20}}{m_{2}}-\frac{K_{10}}{m_{1}}+\frac{K_{12}}{m_{2}}-\frac{K_{12}}{m_{1}}=0,$$

(5b) $$\frac{c_{2}}{m_{2}}-\frac{c_{1}}{m_{1}}=0.$$

Equations (5a) and (5b) tell us that the balance tuning actually includes stiffness tuning and damping coefficient tuning. Since the mass of the test masses can be easily matched to 1 ppm, we can neglect the minute difference in mass tentatively, and the balance conditions are simplified as *K*_10_ = *K*_20_ and *c*_1_ = *c*_2_. In the SGGs, stiffness coefficients are determined by parameters of the levitating coils, the supercurrents in the coils and the parameters of the superconducting circuit (refer to the attached Appendix A). Typically, they are at the order of magnitude of 10^3^ N/m. Limited by the state of the art of the fabrication techniques, it is difficult to reduce the difference in the parameters of the paired coils to less than 1%. On the other hand, the residual gas collision is the only damping mechanism for superconducting oscillators in a vacuum chamber. The damping coefficient is generally small. The typical value is at the order of magnitude of 10^−3^ N/(m/s). This sets an upper limit for the mismatch of the damping coefficients. Even at the upper limit, the mismatch of ωc can be estimated to be smaller than that of the stiffness coefficients by about two orders of magnitude within the concerning frequency band (0.001–50 Hz). In fact, the paired oscillators are designed to be the same in geometric shape and are installed in the same vacuum chamber, so the mismatch of damping coefficient can be expected to be small. Keeping the above facts in mind, one can immediately realize that stiffness tuning dominates damping tuning, at least in the rough tuning stage.

As mentioned previously, the stiffness coefficients can be tuned by injecting an adjusting current into the superconducting circuit that is associated with the levitating coils. The tuning principle is illustrated in Appendix A. The key point in stiffness tuning is to find a reliable criterion to guide the tuning operation. For an SGG, it is impossible to directly measure *K*_10_, *K*_20_, or the difference between them, but the displacements of two test masses can be easily monitored. Keeping this in mind, one would naturally think of whether an analysis of the motion of the test masses would provide a useful guide to the tuning. Indeed, Equation (3) implies a positive answer to the question.

When acceleration is applied to the instrument, the amplitude–frequency response functions of the displacement of two test masses, which are denoted by $H_{1}^{c}\left(\omega \right)$ and $H_{2}^{c}\left(\omega \right)$, respectively, can be derived from Equation (3):

(6a) $$\left|H_{1}^{c}\left(\omega \right)\right|=\left|\frac{x_{1}\left(\omega \right)}{a_{c}\left(\omega \right)}\right|=\frac{\sqrt{{\left(\omega^{2}-\frac{K_{20}}{m_{2}}+\frac{K_{12}}{m_{1}}\right)}^{2}+{\left(\omega \frac{c_{2}}{m_{2}}\right)}^{2}}}{\sqrt{{\left(\omega^{4}-\left(\frac{K_{10}}{m_{1}}+\frac{K_{20}}{m_{2}}+\frac{c_{1}c_{2}}{m_{1}m_{2}}\right)\omega^{2}+\frac{K_{10}K_{20}}{m_{1}m_{2}}-\frac{{K_{12}}^{2}}{m_{1}m_{1}}\right)}^{2}+{\left(\omega^{3}\left(\frac{c_{1}}{m_{1}}+\frac{c_{2}}{m_{2}}\right)+\omega \left(\frac{c_{1}K_{20}}{m_{1}m_{2}}+\frac{K_{10}c_{2}}{m_{1}m_{2}}\right)\right)}^{2}}},$$

(6b) $$\left|H_{2}^{c}\left(\omega \right)\right|=\left|\frac{x_{2}\left(\omega \right)}{a_{c}\left(\omega \right)}\right|=\frac{\sqrt{{\left(\omega^{2}-\frac{K_{10}}{m_{1}}+\frac{K_{12}}{m_{2}}\right)}^{2}+{\left(\omega \frac{c_{1}}{m_{1}}\right)}^{2}}}{\sqrt{{\left(\omega^{4}-\left(\frac{K_{10}}{m_{1}}+\frac{K_{20}}{m_{2}}+\frac{c_{1}c_{2}}{m_{1}m_{2}}\right)\omega^{2}+\frac{K_{10}K_{20}}{m_{1}m_{2}}-\frac{{K_{12}}^{2}}{m_{1}m_{1}}\right)}^{2}+{\left(\omega^{3}\left(\frac{c_{1}}{m_{1}}+\frac{c_{2}}{m_{2}}\right)+\omega \left(\frac{c_{1}K_{20}}{m_{1}m_{2}}+\frac{K_{10}c_{2}}{m_{1}m_{2}}\right)\right)}^{2}}}.$$

**Figure 1:**
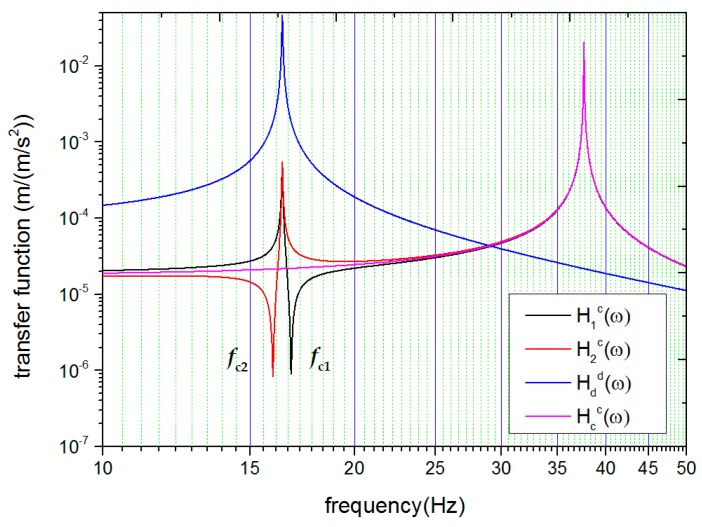
Four calculated frequency response functions: the displacements of individual test mass upon platform acceleration $\left|H_{1}^{c}\left(\omega \right)\right|$ and $\left|H_{2}^{c}\left(\omega \right)\right|$ when the damping coefficient is assumed to be 0.001 N/(m/s) and the mismatch of stiffness is 5%, the common-mode displacement $\left|H_{c}^{c}\left(\omega \right)\right|$ and differential-mode displacement $\left|H_{d}^{d}\left(\omega \right)\right|$ when all of the parameters are ideally matched. Characteristic frequencies $f_{c1}$ and $f_{c2}$ will appear in $\left|H_{1}^{c}\left(\omega \right)\right|$ and $\left|H_{2}^{c}\left(\omega \right)\right|$ when stiffness is mismatched.

These functions are plotted in Figure 1 with typical parameters. In the case that all the parameters are ideally matched, the motion of the system can be decomposed into two normal modes: the common mode and the differential mode. The amplitude–frequency response functions of these two motion modes are given with a set of generalized coordinates $x_{d}=x_{1}-x_{2}$ and $x_{c}=(x_{1}+x_{2})/2$:

(7a) $$\left|H_{c}^{c}\left(\omega \right)\right|=\left|\frac{x_{c}\left(\omega \right)}{a_{c}\left(\omega \right)}\right|=\frac{1}{2\sqrt{{\left(\omega^{2}-\left(\frac{K_{10}}{m_{1}}+\frac{K_{12}}{m_{1}}\right)\right)}^{2}+{\left(\omega \frac{c_{1}}{m_{1}}\right)}^{2}}}$$

(7b) $$\left|H_{d}^{d}\left(\omega \right)\right|=\left|\frac{x_{d}\left(\omega \right)}{a_{d}\left(\omega \right)}\right|=\frac{1}{\sqrt{{\left(\omega^{2}-\left(\frac{K_{10}}{m_{1}}-\frac{K_{12}}{m_{1}}\right)\right)}^{2}+{\left(\omega \frac{c_{1}}{m_{1}}\right)}^{2}}}.$$

To make things clearer, these two functions are also plotted in Figure 1.

**Figure 2:**
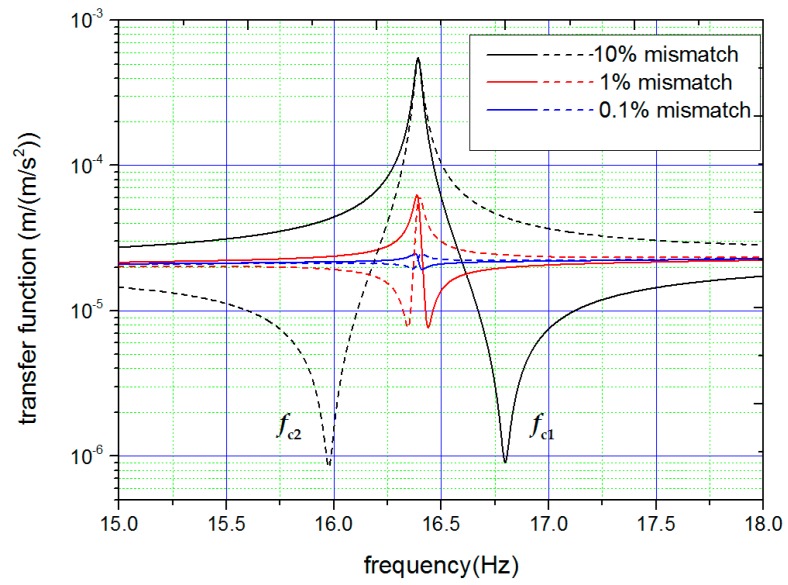
Calculated frequency response functions of displacement for each test mass at different mismatch of stiffness. The solid and dashed lines are used to distinguish the different test masses. When the mismatch of stiffness becomes smaller, both the difference between $f_{c1}$ and $f_{c2}$ and the depth of the valley keep reducing.

One can find a number of interesting things in Figure 1. When the stiffness parameters of the oscillators are ideally matched, $H_{1}^{c}\left(\omega \right)$, $H_{2}^{c}\left(\omega \right)$, and $H_{c}^{c}\left(\omega \right)$ share the same function, and present only one resonance frequency $f_{c}^{c}$ on the high-frequency side. $f_{c}^{c}$ is called the common-mode resonance frequency. However, in cases where the stiffness parameters are not matched, $H_{1}^{c}\left(\omega \right)$ and $H_{2}^{c}\left(\omega \right)$ split into different forms, and additional resonance peaks appear at the differential-mode resonance frequency (refer to the curve of $H_{d}^{d}\left(\omega \right)$ in Figure 1). Accompanying this resonance peak is a nearby valley. The emergence of the valleys allows us to define two characteristic frequencies, $f_{c1}$ and $f_{c2}$, at which $H_{1}^{c}\left(\omega \right)$ and $H_{2}^{c}\left(\omega \right)$ show the minimum response to platform acceleration respectively. $f_{c1}$ is always different from $f_{c2}$ if the stiffness parameters are not ideally matched. When the stiffness parameters become better matched, both the difference between $f_{c1}$ and $f_{c2}$ and the depth of the valley is reduced, as shown in Figure 2. The above findings clearly indicate (i) that the appearance of the features around the differential-mode resonance frequency can be used itself as the criterion to check if the stiffness parameters are matched and (ii) that the difference between the characteristic frequencies can be used to estimate the amount of mismatch and guide the tuning operation.

Consequently, a scheme to tune the stiffness parameters emerges. In the scheme, the displacement responses of the two test masses to common-mode acceleration are measured simultaneously as functions of frequency. The characteristic frequencies $f_{c1}$ and $f_{c2}$ are determined by the measurement for each test mass. By comparing the characteristic frequency with the differential-mode resonance frequency, one can make a judgment as to whether the stiffness parameter associated with this test mass should be adjusted to become larger or smaller. Furthermore, using the designed parameters of the superconducting circuit, the magnitude of the adjusting current can be roughly calculated according to the measured value of $f_{c1}{}^{2}-f_{c2}{}^{2}$. Guided by the above information, the stiffness parameters are tuned for the first turn. The procedure can be repeated until the measurement of response function cannot provide any further useful information. The tuning limit is thereby approached.

The underlying theoretic basis for the tuning scheme will be more intuitive if the damping effect is neglected. In this case, Equations (6a) and (6b) become

(8a) $$\left|H_{1}^{c}\left(\omega \right)\right|=\left|\frac{x_{1}\left(\omega \right)}{a_{c}\left(\omega \right)}\right|=\frac{\omega^{2}-\frac{K_{20}}{m_{2}}+\frac{K_{12}}{m_{1}}}{\omega^{4}-\left(\frac{K_{10}}{m_{1}}+\frac{K_{20}}{m_{2}}\right)\omega^{2}+\frac{K_{10}K_{20}}{m_{1}m_{2}}-\frac{K_{12}{}^{2}}{m_{1}m_{1}}}$$

(8b) $$\left|H_{2}^{c}\left(\omega \right)\right|=\left|\frac{x_{2}\left(\omega \right)}{a_{c}\left(\omega \right)}\right|=\frac{\omega^{2}-\frac{K_{10}}{m_{1}}+\frac{K_{12}}{m_{2}}}{\omega^{4}-\left(\frac{K_{10}}{m_{1}}+\frac{K_{20}}{m_{2}}\right)\omega^{2}+\frac{K_{10}K_{20}}{m_{1}m_{2}}-\frac{K_{12}{}^{2}}{m_{1}m_{1}}}.$$

Assuming the test masses are reasonably identical, the displacement response now vanishes at the frequency of $f_{ci}$ (*i* = 1 and 2). The $f_{c1}{}^{2}-f_{c2}{}^{2}$ will be a direct measurement of the mismatch of stiffness.

**Figure 3:**
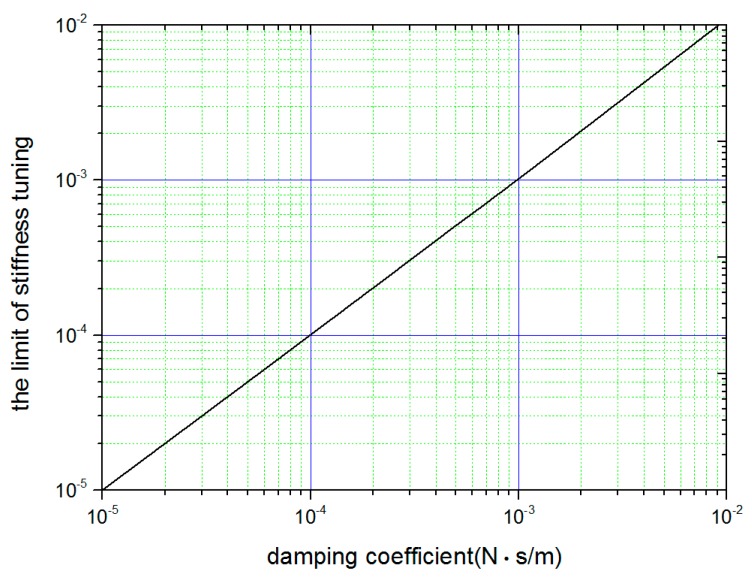
Relationship of the damping coefficient and the limit of stiffness balance. The amplitude of the response function at differential-mode frequency was 2 × 10^−5^ (m/m/s^2^), and the measurement resolution was set as 1 × 10^−6^ (m/m/s^2^) artificially. For each damping coefficient, the depth of the valley around $f_{ci}$ of the response function was calculated at different mismatches of stiffness to find the limit of stiffness balance.

For a real SGG, damping, albeit slight, inevitably exists. The damping effect on the tuning accuracy should be evaluated. Noticing that the depth of the valley around $f_{ci}$ on the curve of the response function will diminish with the improved matching state, the final accuracy can be determined by finding the mismatch under which the valley depth is comparable with the measurement resolution of the response functions. In this way, the damping effect on the tuning accuracy was investigated, and a typical result is plotted in Figure 3. Obviously, the tuning accuracy was high when the damping coefficient was small. In [8], the damping coefficient of the SGG was as low as 10^−4^ N/(m/s), and the tuning accuracy could reach 10^−4^ with this scheme.

## 3. Experimental Test

**Figure 4:**
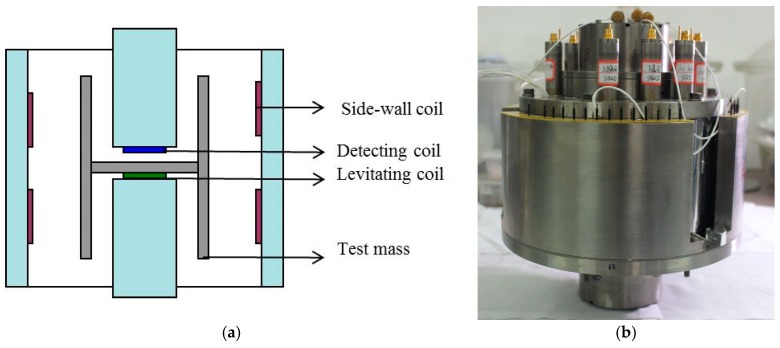
(**a**) Cross sectional illustration showing the layout of different types of coils; (**b**) the assembled superconducting gravity gradiometer (SGG) sensor.

The stiffness tuning scheme was tested on a T_zz_ single-component SGG, which is under development in our group. Figure 4a illustrates the sensor structure. The niobium test masses were designed as a cylinder with a division plate inside following the Arkex design [7]. The mass of two test masses was balanced to 1 ppm. The levitating coils were placed under the division plate and the displacement detecting coils over them. Furthermore, four pairs of coils were installed adjacent to the outside wall of each test mass, which were used to hold the test-mass attitude.

**Figure 5:**
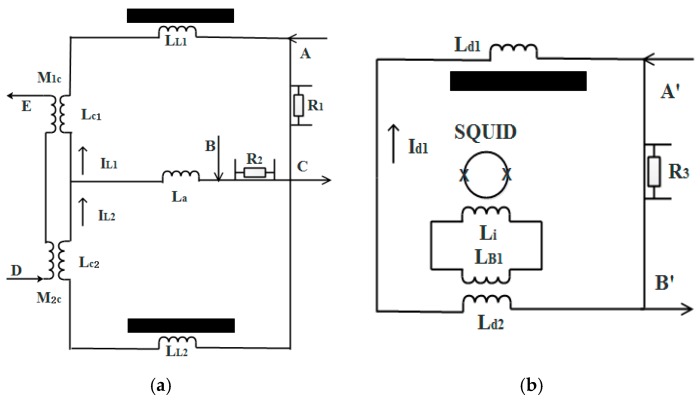
Superconducting circuits for the sensitive degree of freedom. (**a**) The levitating circuit and (**b**) the test mass displacement detecting circuit. The heat-switches R_1_ and R_2_ and the current injecting ports A, B, and C are used to inject and adjust the levitating currents; mutual inductions M_1c_ and M_2c_ and the injecting ports D and E are used to apply a sinusoidal current to produce the modulation acceleration. The heat-switch R_3_ and the current injecting ports A’ and B’ are used to inject the detecting current.

The superconducting circuit for levitating is shown in Figure 5a. Superconducting coupled mutual inductions are added to the outer loop. Through the mutual inductions, a sinusoidal current can be introduced and superposed upon the levitating supercurrent to produce modulation acceleration for both test masses (refer to Appendix A). As the coil parameters are not matched, there is a small difference in the amplitude of the modulation acceleration between the two test masses. However, this difference only modifies the features of the response functions on the high-frequency side, as indicated by Equation (3). These features are not used in the tuning scheme. The method to use a superconducting circuit instead of a shaker to produce common-mode acceleration is favorable for incorporating a self-checking function in the instrument. The displacement of the individual test mass is detected using a typical superconducting circuit as shown in Figure 5b.

**Figure 6:**
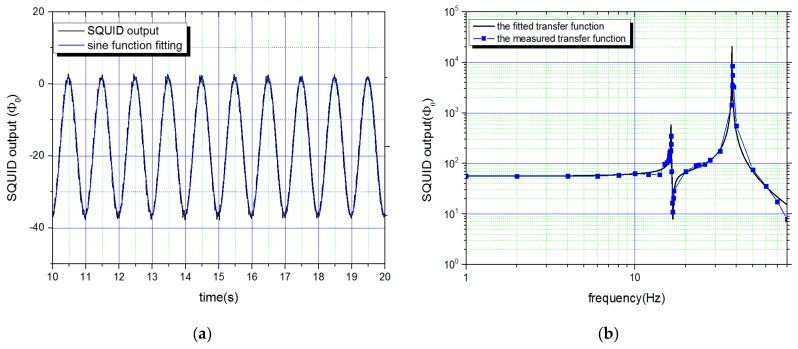
(**a**) The Superconducting Quantum Interference Device output of one test mass under 1 Hz modulation acceleration. The output signal is fitted with a sine function to determine the amplitude. (**b**) The measured and fitted transfer function. The common and differential-modes natural frequencies and the characteristic frequency were all detected, and the characteristic frequency was obtained by the fitted transfer function.

The SGG sensor was installed in a vacuum chamber with a residual helium pressure of about 10 Pa. After the system was cooled to 4.2 K, a persistent current of 6.8 A was injected into the levitating coils. The current of the sensing coils was set to 10 mA. The displacement of the test masses was transferred to the Superconducting Quantum Interference Device (SQUID)’s output by the sensing circuit. To obtain a common-mode agitating acceleration, a sinusoidal current with a constant amplitude of 20 mA was applied to the second winding of the mutual inductance by a current source meter. The produced modulation acceleration was roughly estimated to be 0.01 m/s^2^ in amplitude. The sinusoidal motion of the test mass could be immediately monitored as shown in Figure 6a. By fitting the data with a sine function, the displacement amplitude was determined for each test mass at the given frequency. Since the scale factors for displacement sensing were not calibrated, the displacement amplitude was simply denoted by the SQUID output. Keeping the amplitude of the agitating current constant, the displacement amplitude of each test mass was measured at varied frequencies. The collected data was fitted with the formula presented in Equation (6), and the characteristic frequencies ($f_{ci}$) were subsequently obtained (Figure 6b).

**Figure 7:**
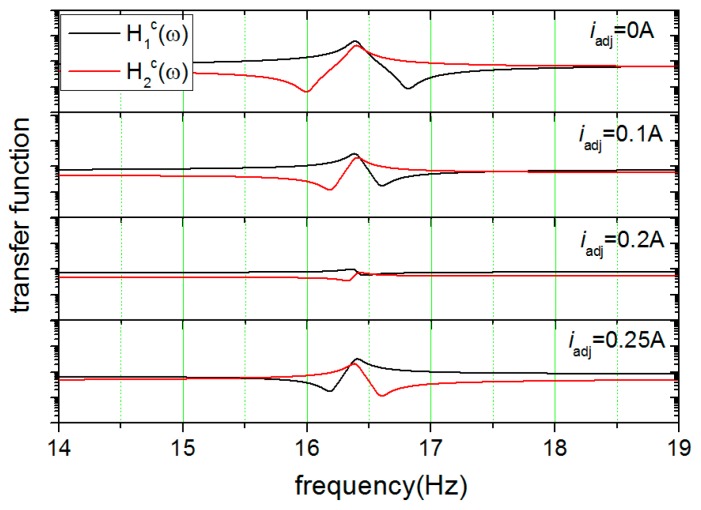
The measured displacement response functions under different adjusting currents.

In the experiment, the first turn of the measurement of the response functions yielded $f_{c1}=16.0\;\mathrm{Hz}$ and $f_{c2}=16.8\;\mathrm{Hz}$, and the mismatch of stiffness, defined as $\left(f_{c1}{}^{2}-f_{c2}{}^{2}\right)/f_{c1}{}^{2}$, was calculated to be 0.1 for the initial state. In the first turn of tuning, a test current of 0.1 A was injected into the levitating circuit so that the currents in the levitating coils were adjusted to 6.85 A and 6.75 A, respectively. This turn of tuning provided a pair of new characteristic frequencies, which were $f_{c1}=16.2\;\mathrm{Hz}$ and $f_{c2}=16.6\;\mathrm{Hz}$. The mismatch of stiffness was now reduced to 0.05. With the obtained results, an adjusting current of 0.21 A was calculated and applied in the second turn of tuning, resulting in a pair of much closer characteristic frequencies ($f_{c1}=16.47\;\mathrm{Hz}$ and $f_{c2}=16.53\;\mathrm{Hz}$). The mismatch was further reduced to 5 × 10^−3^. Furthermore, the adjusting current was set to 0.25 A deliberately to see the over-tuning results. In this case, the appearance sequence with increasing frequency of the valley and the peak on the curves changed for each test mass, and the absolute value of the difference between the two characteristic frequencies increased. The measured displacement response functions under different adjusting currents are shown in Figure 7. To explore the balance limit of the scheme, a total of five turns of tuning were carried out. The experimental results showed that the stiffness parameters could be balanced quite close to the limit by the first two turns of tuning. The mismatch of stiffness was finally reduced to 10^−3^.

## 4. Conclusions

Based on a systematic theoretical analysis, a new tuning scheme was experimentally tested for the stiffness balance of an SGG. In this scheme, characteristic frequencies are adopted as the tuning criterion. The criterion did not depend on the mismatch of the scale factors for displacement detection, so stiffness tuning could be conducted independently. This was of great advantage in terms of tuning the full-band common-mode balance. However, the criterion would only work if the damping coefficient was relatively small and the mass of the oscillators had already been balanced at a relatively high accuracy. Otherwise, the tuning accuracy was limited by the effects induced by these two factors rather than the measurement resolution of the characteristic frequencies.

## Appendix A. Analysis of the Superconducting Circuits

### Appendix A.1. Stiffness Parameters and Their Adjustments

The superconducting circuits of the sensitive degree of freedom are illustrated in Figure 5. The interaction between the current-carrying coil and the test mass in the Meissner state can be described by the test mass’s displacement dependence of the coil induction [9]. This relationship can be approximated by

(A1) $$\left\{ \begin{array}{l} L_{L1}\left(x_{1}\right)=L_{L10}\left(1+\lambda_{L10}x_{1}\right)\\ L_{L2}\left(x_{2}\right)=L_{L20}\left(1+\lambda_{L20}x_{2}\right) \end{array}\right.$$

where *L**_Li_*_0_ (*i* = 1, 2) is the inductance of the coils when test masses stay at equilibrium position; $\lambda_{Li0}$ (i = 1, 2) is the linear coefficient of each coil.

For the levitating circuit, the conservation law of flux for superconducting loop requires [10]

(A2) $$\left\{ \begin{array}{l} \left(L_{L1}\left(x_{1}\right)+L_{c1}\right)I_{L1}\left(x_{1},x_{2}\right)+L_{a}\left(I_{L1}\left(x_{1},x_{2}\right)-I_{L2}\left(x_{1},x_{2}\right)\right)=\left(L_{L10}+L_{c1}\right)I_{L10}+L_{a}\left(I_{L10}-I_{L20}\right)\\ \left(L_{L2}\left(x_{1}\right)+L_{c2}\right)I_{L2}\left(x_{1},x_{2}\right)-L_{a}\left(I_{L1}\left(x_{1},x_{2}\right)-I_{L2}\left(x_{1},x_{2}\right)\right)=\left(L_{L20}+L_{c2}\right)I_{L20}-L_{a}\left(I_{L10}-I_{L20}\right) \end{array}\right.$$

where *I_L_*_1_(*x*_1_,*x*_2_) and *I_L_*_2_(*x*_1_,*x*_2_) are the supercurrents in the levitating coils. *L*_c1_ and *L*_c2_ are the inductances of primary windings of the modulation acceleration agitating transformers.

Solving Equation (A2) for *I_L_*_1_(*x*_1_,*x*_2_) and *I_L_*_2_(*x*_1_,*x*_2_) yields

(A3) $$\left\{ \begin{array}{l} I_{L1}\left(x_{1},x_{2}\right)=\frac{\left(L_{L10}+L_{c1}\right)I_{L10}\left(\left(L_{L2}\left(x_{2}\right)+L_{c2}\right)+L_{a}\right)+\left(L_{L20}+L_{c2}\right)I_{L20}L_{a}}{\left(\left(L_{L1}\left(x_{1}\right)+L_{c1}\right)\left(L_{L2}\left(x_{2}\right)+L_{c2}\right)+L_{a}\left(\left(L_{L1}\left(x_{1}\right)+L_{c1}\right)+\left(L_{L2}\left(x_{2}\right)+L_{c2}\right)\right)\right)}\\ I_{L2}\left(x_{1},x_{2}\right)=\frac{\left(L_{L20}+L_{c1}\right)I_{L20}\left(\left(L_{L1}\left(x_{1}\right)+L_{c1}\right)+L_{a}\right)+\left(L_{L10}+L_{c1}\right)I_{L10}L_{a}}{\left(\left(L_{L1}\left(x_{1}\right)+L_{c1}\right)\left(L_{L2}\left(x_{2}\right)+L_{c2}\right)+L_{a}\left(\left(L_{L1}\left(x_{1}\right)+L_{c1}\right)+\left(L_{L2}\left(x_{2}\right)+L_{c2}\right)\right)\right)} \end{array}\right..$$

The magnetic force terms arising from the levitating circuits are derived to first order as [12]

(A4) $$\left(\begin{array}{l} F_{L1}\\ F_{L2} \end{array}\right)=\left(\begin{array}{l} \frac{1}{2}\frac{\partial L_{L1}}{x_{1}}I_{L1}{}^{2}\\ \frac{1}{2}\frac{\partial L_{L2}}{x_{2}}I_{L2}{}^{2} \end{array}\right)=\left(\begin{array}{l} F_{L10}\\ F_{L20} \end{array}\right)-\left(\begin{array}{l} K_{L10}\;K_{L12}\\ K_{L21}\;K_{L20} \end{array}\right)\left(\begin{array}{l} x_{1}\\ x_{2} \end{array}\right)$$

where

(A5) $$\left\{ \begin{array}{l} F_{L10}=\frac{1}{2}L_{L10}\lambda_{L10}I_{L10}{}^{2}\\ F_{L20}=\frac{1}{2}L_{L20}\lambda_{L20}I_{L20}{}^{2} \end{array}\right.$$

are the DC forces, which counterbalance the gravity of the test masses, and

(A6) $$\left\{ \begin{array}{l} K_{L10}=\frac{L_{L10}{}^{2}\lambda_{L10}{}^{2}\left(L_{a}+L_{L20}+L_{c2}\right)I_{L10}{}^{2}}{\left(\left(L_{L10}+L_{c1}\right)\left(L_{L20}+L_{c2}\right)+L_{a}\left(\left(L_{L10}+L_{c1}\right)+\left(L_{L20}+L_{c2}\right)\right)\right)}\\ K_{L20}=\frac{L_{L20}{}^{2}\lambda_{L20}{}^{2}\left(L_{a}+L_{L10}+L_{c1}\right)I_{L20}{}^{2}}{\left(\left(L_{L10}+L_{c1}\right)\left(L_{L20}+L_{c2}\right)+L_{a}\left(\left(L_{L10}+L_{c1}\right)+\left(L_{L20}+L_{c2}\right)\right)\right)}\\ K_{L12}=\frac{L_{L10}\lambda_{L10}L_{L20}\lambda_{L20}L_{a}I_{L10}I_{L20}}{\left(\left(L_{L10}+L_{c1}\right)\left(L_{L20}+L_{c2}\right)+L_{a}\left(\left(L_{L10}+L_{c1}\right)+\left(L_{L20}+L_{c2}\right)\right)\right)}\\ K_{L21}=\frac{L_{L10}\lambda_{L10}L_{L20}\lambda_{L20}L_{a}I_{L10}I_{L20}}{\left(\left(L_{L10}+L_{c1}\right)\left(L_{L20}+L_{c2}\right)+L_{a}\left(\left(L_{L10}+L_{c1}\right)+\left(L_{L20}+L_{c2}\right)\right)\right)} \end{array}\right.$$

are the stiffness coefficients associated with the levitating circuits.

The stiffness coefficients are directly determined by parameters of the levitating coils, the levitating currents in the coils, and the parameters of the superconducting circuit.

For the stiffness balancing, *K_L_*_10_ and *K_L_*_20_ must be identical, which yields

(A7) $$L_{L10}{}^{2}\lambda_{L10}{}^{2}\left(L_{a}+L_{L20}+L_{c2}\right)I_{L10}{}^{2}=L_{L20}{}^{2}\lambda_{L20}{}^{2}\left(L_{a}+L_{L10}+L_{c1}\right)I_{L20}{}^{2}.$$

This condition can be satisfied by adjusting *I_L_*_10_ and *I_L_*_20_ to

(A8) $$\frac{I_{L10}{}^{2}}{I_{L20}{}^{2}}=\frac{L_{L20}{}^{2}\lambda_{L20}{}^{2}\left(L_{a}+L_{L10}+L_{c1}\right)}{L_{L10}{}^{2}\lambda_{L10}{}^{2}\left(L_{a}+L_{L20}+L_{c2}\right)}.$$

To adjust the levitating currents, an adjusting current *i_a_* is injected into the middle arm using the heat-switch R_2_. Then, the levitating currents in the coils will change to

(A9) $$\left\{ \begin{array}{l} I_{L10}=I_{L0}+\frac{i_{a}}{2}\\ I_{L20}=I_{L0}-\frac{i_{a}}{2} \end{array}\right..$$

### Appendix A.2. Test Mass Displacement Detection

In the displacement detecting circuit, the relationship between the inductance of the detecting coil and the test mass displacement is

(A10) $$L_{d1}\left(x_{1}\right)=L_{d10}\left(1-\lambda_{d10}x_{1}\right).$$

Then, the conservation of flux in the detecting circuit yields

(A11) $$\left(L_{d1}\left(x_{1}\right)+L_{B}\right)I_{d1}\left(x_{1}\right)=\left(L_{d10}+L_{B}\right)I_{d10}$$

where *I**_d_*_10_ is the initial detecting current stored in the circuit, and *L_B_* is the effective inductance of the transformer coupled to the SQUID, which is given by

(A12) $$L_{B}=L_{B1}-\frac{M_{B}{}^{2}}{L_{B2}+L_{i}}$$

where *L**_B_*_1_ and *L**_B_*_2_ are the primary and secondary inductance of the transformer, *M**_B_* is the mutual inductance, and *L_i_* is the inductance of the SQUID input coil.

Combining Equations (A10) and (A11), *I_d_*_1_(*x*_1_) can be solved to first order as

(A13) $$I_{d1}\left(x_{1}\right)=I_{d10}+\frac{L_{d10}\lambda_{d10}I_{d10}}{L_{d10}+L_{B}}x_{1}.$$

This current is proportional to the test mass displacement and will be amplified and transformed into voltage signal by the SQUID.

### Appendix A.3. Modulation Acceleration Produced by Levitating Circuit

When a small current is injected by the modulation acceleration agitating transformers, the flux coupled by the mutual inductions enters the levitating circuit and the currents, which are denoted as *I_L_*_1_′(*x*_1_,*x*_2_) and *I_L_*_2_′(*x*_1_,*x*_2_), in the levitating coils will be changed. Now, the equation of flux conservation in the circuit changes to

(A14) $$\left\{ \begin{array}{l} \left(L_{L1}\left(x_{1}\right)+L_{c1}\right)I_{L1}\prime \left(x_{1},x_{2}\right)+L_{a}\left(I_{L1}\prime \left(x_{1},x_{2}\right)-I_{L2}\prime \left(x_{1},x_{2}\right)\right)=\left(L_{L10}+L_{c1}\right)I_{L10}+L_{a}\left(I_{L10}-I_{L20}\right)+M_{c1}i_{c}\\ \left(L_{L2}\left(x_{1}\right)+L_{c2}\right)I_{L2}\prime \left(x_{1},x_{2}\right)-L_{a}\left(I_{L1}\prime \left(x_{1},x_{2}\right)-I_{L2}\prime \left(x_{1},x_{2}\right)\right)=\left(L_{L20}+L_{c2}\right)I_{L20}-L_{a}\left(I_{L10}-I_{L20}\right)+M_{c2}i_{c}\\ \left(L_{L1}\left(x_{1}\right)+L_{c1}\right)I_{L1}\prime \left(x_{1},x_{2}\right)+\left(L_{L2}\left(x_{1}\right)+L_{c2}\right)I_{L2}\prime \left(x_{1},x_{2}\right)=\left(L_{L10}+L_{c1}\right)I_{L10}+\left(L_{L20}+L_{c2}\right)I_{L20}+M_{c1}i_{c}+M_{c2}i_{c} \end{array}\right.$$

where *i**_c_* is the injected current, and *M**_c_*_1_ and *M**_c_*_2_ are the mutual inductance of the two transformers respectively.

*I_L_*_1_’(*x*_1_,*x*_2_) and *I_L_*_2_’(*x*_1_,*x*_2_) can be solved as

(A15) $$\left\{ \begin{array}{l} I_{L1}\prime \left(x_{1},x_{2}\right)=I_{L1}\left(x_{1},x_{2}\right)+\frac{\left(\left(L_{L20}+L_{a}+L_{c2}\right)M_{c1}+L_{a}M_{c2}\right)i_{c}}{\left(\left(L_{L10}+L_{c1}\right)\left(L_{L20}+L_{c2}\right)+L_{a}\left(\left(L_{L10}+L_{c1}\right)+\left(L_{L20}+L_{c2}\right)\right)\right)}\\ I_{L2}\prime \left(x_{1},x_{2}\right)=I_{L2}\left(x_{1},x_{2}\right)+\frac{\left(\left(L_{L10}+L_{a}+L_{c1}\right)M_{c2}+L_{a}M_{c1}\right)i_{c}}{\left(\left(L_{L10}+L_{c1}\right)\left(L_{L20}+L_{c2}\right)+L_{a}\left(\left(L_{L10}+L_{c1}\right)+\left(L_{L20}+L_{c2}\right)\right)\right)} \end{array}\right..$$

The applied current introduces a new current item that is proportional to *i**_c_* and not related to the test mass displacement. The total magnetic forces produced by the levitating coils are recalculated to first order as

(A16) $$\left(\begin{array}{l} F_{L1}\\ F_{L2} \end{array}\right)=\left(\begin{array}{l} \frac{1}{2}\frac{\partial L_{L1}}{x_{1}}I_{L1}\prime^{2}\\ \frac{1}{2}\frac{\partial L_{L2}}{x_{2}}I_{L2}\prime^{2} \end{array}\right)=\left(\begin{array}{l} F_{L10}\\ F_{L20} \end{array}\right)-\left(\begin{array}{l} K_{L10}\;K_{L12}\\ K_{L12}\;K_{L20} \end{array}\right)\left(\begin{array}{l} x_{1}\\ x_{2} \end{array}\right)+\left(\begin{array}{l} F_{c1}\\ F_{c2} \end{array}\right)$$

where

(A17) $$\left(\begin{array}{l} F_{c1}\\ F_{c2} \end{array}\right)=\left(\begin{array}{l} \frac{L_{L10}\lambda_{L10}I_{L10}\left(\left(L_{L20}+L_{a}+L_{c2}\right)M_{c1}+L_{a}M_{c2}\right)i_{c}}{\left(\left(L_{L10}+L_{c1}\right)\left(L_{L20}+L_{c2}\right)+L_{a}\left(\left(L_{L10}+L_{c1}\right)+\left(L_{L20}+L_{c2}\right)\right)\right)}\\ \frac{L_{L20}\lambda_{L20}I_{L20}\left(\left(L_{L10}+L_{a}+L_{c1}\right)M_{c2}+L_{a}M_{c1}\right)i_{c}}{\left(\left(L_{L10}+L_{c1}\right)\left(L_{L20}+L_{c2}\right)+L_{a}\left(\left(L_{L10}+L_{c1}\right)+\left(L_{L20}+L_{c2}\right)\right)\right)} \end{array}\right)$$

are the modulation forces introduced by the applied current *i**_c_*. The accelerations applied to each test mass are

(A18) $$\left\{ \begin{array}{l} a_{1}=\frac{F_{c1}}{m_{1}}\\ a_{2}=\frac{F_{c2}}{m_{2}} \end{array}\right..$$
